# First-in-class candidate therapeutics that target mitochondria and effectively prevent cancer cell metastasis: mitoriboscins and TPP compounds

**DOI:** 10.18632/aging.103336

**Published:** 2020-05-24

**Authors:** Béla Ózsvári, Federica Sotgia, Michael P. Lisanti

**Affiliations:** 1 Translational Medicine, School of Science, Engineering and Environment (SEE), University of Salford, Greater Manchester, United Kingdom

**Keywords:** cancer stem-like cells (CSCs), mitochondrial inhibitors, treatment failure, breast cancer, metastasis prophylaxis

## Abstract

Cancer stem cells (CSCs) have been proposed to be responsible for tumor recurrence, distant metastasis and drug-resistance, in the vast majority of cancer patients. Therefore, there is an urgent need to identify new drugs that can target and eradicate CSCs. To identify new molecular targets that are unique to CSCs, we previously compared MCF7 2D-monolayers with 3D-mammospheres, which are enriched in CSCs. We observed that 25 mitochondrial-related proteins were >100-fold over-expressed in 3D-mammospheres. Here, we used these 25 proteins to derive short gene signatures to predict distant metastasis (in N=1,395 patients) and tumor recurrence (in N=3,082 patients), by employing a large collection of transcriptional profiling data from ER(+) breast cancer patients. This analysis resulted in a 4-gene signature for predicting distant metastasis, with a hazard ratio of 1.91-fold (P=2.2e-08). This provides clinical evidence to support a role for CSC mitochondria in metastatic dissemination. Next, we employed a panel of mitochondrial inhibitors, previously shown to target mitochondria and selectively inhibit 3D-mammosphere formation in MCF7 cells and cell migration in MDA-MB-231 cells. Remarkably, these five mitochondrial inhibitors had only minor effects or no effect on MDA-MB-231 tumor formation, but preferentially and selectively inhibited tumor cell metastasis, without causing significant toxicity. Mechanistically, all five mitochondrial inhibitors have been previously shown to induce ATP-depletion in cancer cells. Since 3 of these 5 inhibitors were designed to target the large mitochondrial ribosome, we next interrogated whether genes encoding the large mitochondrial ribosomal proteins (MRPL) also show prognostic value in the prediction of distant metastasis in both ER(+) and ER(-) breast cancer patients. Interestingly, gene signatures composed of 6 to 9 MRPL mRNA-transcripts were indeed sufficient to predict distant metastasis, tumor recurrence and Tamoxifen resistance. These gene signatures could be useful as companion diagnostics to assess which patients may benefit most from anti-mito-ribosome therapy. Overall, our studies provide the necessary proof-of-concept, and *in vivo* functional evidence, that mitochondrial inhibitors can successfully and selectively target the biological process of cancer cell metastasis. Ultimately, we envision that mitochondrial inhibitors could be employed to develop new treatment protocols, for clinically providing metastasis prophylaxis, to help prevent poor clinical outcomes in cancer patients.

## INTRODUCTION

Today, breast cancer treatment requires a multi-disciplinary approach, involving an extensive medical team consisting of specialized surgeons, medical oncologists, oncology nurses, as well as radiologists and radiology technicians, to perform anti-cancer therapy, which consists of tumor excision, chemo- or hormonal-therapy, as well as radiation therapy. Despite these major advances, many patients still ultimately undergo treatment failure, in the form of tumor recurrence and distant metastasis. Unfortunately, distant metastasis causes premature death, in >90% of cancer patients with treatment failure [1–5]. Therefore, there is a clear need to develop new strategies to prevent cancer cell metastasis.

Local and distant metastases are thought to be caused by a small sub-population of cancer cells, known as cancer stem cells (CSCs) [1–5]. These CSCs are unique, in the sense that they can regenerate tumors in immune-deficient mice, as xenografts, and they undergo anchorage-independent proliferation and the EMT, allowing them to disseminate throughout the body, thereby creating local and distant metastatic lesions, which are largely chemo- and radio-therapy resistant [1–5]. However, it remains largely unknown, what are the precise vulnerabilities of CSCs.

Recently, we identified cancer cell mitochondria as a new promising therapeutic target for the eradication of CSCs [6–8]. New evidence suggests that CSCs have elevated levels of mitochondrial biogenesis, that helps to energetically drive their rapid propagation and anchorage-independent growth [6–10]. In support of this notion, metastatic breast cancer cells in positive lymph nodes, removed from patients, show a significant increase in mitochondrial Complex IV activity, as seen by histochemical- and immuno-staining [11, 12].

Importantly, mitochondrial biogenesis is strictly dependent on the function of the mitochondrial ribosome, which consists of both large and small subunits, to effectively carry out the mitochondrial protein translation of 13 key genes that are absolutely required for OXPHOS and mitochondrial ATP production [13]. Interestingly, in eukaryotic cells, mitochondria originally evolved from engulfed aerobic bacteria, an event estimated to have occurred approximately 1.5 billion years ago. Because of this close evolutionary relationship, certain FDA-approved drugs inhibit mitochondrial protein translation as an off-target side effect. For example, Doxycycline (a Tetracycline family member) negatively affects the small mito-ribosome, while Azithromycin (an Erythromycin family member) inhibits the large mito-ribosome. Both Doxycycline and Azithromycin effectively inhibit the anchorage-independent propagation of CSCs, as assessed using the 3D-tumor-sphere assay, in 12 cell lines derived from 8 different cancer types, including breast cancers (MCF7, T47D, MDA-MB-231 and MCF10.DCIS.COM) [13]. Therefore, we proposed that these off-target side-effects could be clinically “re-purposed” as a therapeutic effect.

A recent Phase II clinical trial also showed that Doxycycline treatment (200-mg/day for 2-weeks) of early stage breast cancer patients reduced their CSC tumor load (as assessed by CD44 immuno-staining), between ~17% and ~67%, with a positive response rate approaching nearly 90% [14]. Therefore, inhibition of mitochondrial protein translation may be a new valuable target for eradicating CSCs in patients [14].

To design novel therapeutics to more effectively target the mitochondria, we used the known 3D-structure of the large mammalian mito-ribosome, to perform *in silico* library screening, coupled with phenotypic drug screening, to develop a new family of drug-like compounds, called the Mitoriboscins [15]. Importantly, as predicted, the Mitoriboscins inhibited mitochondrial oxygen consumption rates, resulting in cellular ATP-depletion, and potently inhibited 3D-mammosphere formation, all with an IC-50 in the low micro-molar range [15].

Here, we now show that the Mitoriboscins have only minor effects (23/G4) or no inhibitory effects (24/D4, 24/F9) on tumor growth, but functionally prevent metastatic progression. Quantitatively similar results were obtained with another independent class of mitochondrial inhibitors, namely Butene-1,4-bis-triphenyl-phosphonium (Bis-TPP) and Dodecyl-triphenyl-phosphonium (Dodecyl-TPP) [16, 17]. Bis-TPP and Dodecyl-TPP both contain a TPP moiety, which functions as a chemical signal for mitochondrial targeting [16, 17]. These data provide *in vivo* functional evidence that five mitochondrial inhibitors can successfully and preferentially target the biological process of cancer cell metastasis, without significant toxicity.

## RESULTS

### Cancer stem cell (CSC) based mitochondrial signatures for predicting distant metastasis and tumor recurrence

**Figure 1:**
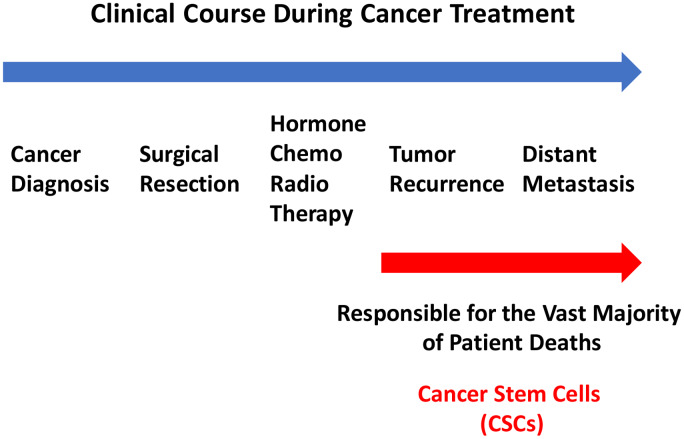
**Clinical course of cancer therapy: Focus on the causes of treatment failure.** After diagnosis, breast cancer patients undergo surgical resection of the primary tumor and then are treated with a specific therapy (hormone/chemo/radio), depending on the breast cancer subtype and clinical staging. However, a significant number of patients ultimately undergo treatment failure, resulting in tumor recurrence and distant metastasis. Distant metastasis is responsible for the premature deaths of >90% of cancer patients, undergoing treatment failure. This phenomenon has been attributed to the propagation and dissemination of CSCs.

After a breast cancer diagnosis, most patients undergo surgical resection of the primary tumor and are then subsequently treated with hormone-, chemo- and/or radio-therapy, depending on the breast cancer subtype. However, many patients ultimately experience treatment failure, resulting in tumor recurrence and distant metastasis. Unfortunately, distant metastasis is responsible for the premature deaths in the vast majority of cancer patients, approaching >90% (Figure 1). Therefore, new diagnostics and therapeutics are urgently needed to prevent and treat metastatic disease, which has been attributed to the existence and resurgence of a small sub-population of cancer cells, known as cancer stem cells (CSCs).

In order to identify new molecular targets that are selectively up-regulated in CSCs, we previously carried out unbiased proteomics analysis on MCF7 cell 2D-monolayers, as directly compared with MCF7 3D-mammospheres, which are known to be highly enriched in CSCs and progenitor cells [6]. As a consequence, we observed that 25 mitochondrial proteins were highly up-regulated by >100-fold, specifically in 3D-mammospheres [6].

**Figure 2:**
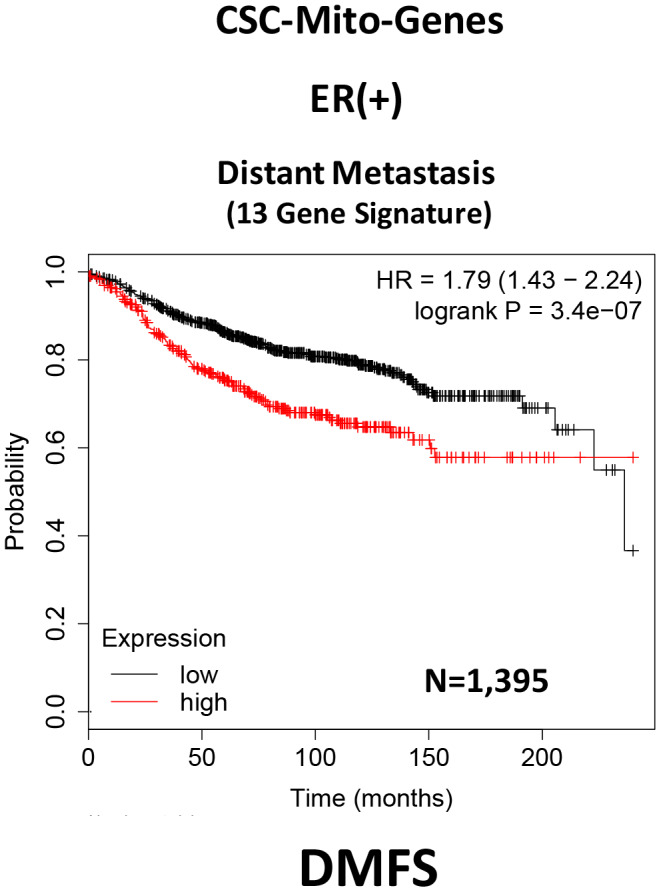
**A CSC-based mitochondrial 13-gene signature predicts distant metastasis in ER(+) breast cancer patients.** We used 13 gene transcripts to create a CSC-based mitochondrial-related gene signature, that effectively predicted distant metastasis in N=1,395 patients (HR=1.79; P=3.4e-07). See also Supplementary Table 1.

Here, we interrogated whether the mRNA transcripts of these mitochondrial proteins show any prognostic value in large numbers of ER(+) human breast cancer patients. Interestingly, we observed that 13 of these 25 gene transcripts showed prognostic value in predicting distant metastasis. We then used these 13 gene transcripts to create a mitochondrial-related gene signature, that effectively predicted distant metastasis in 1,395 patients (HR=1.79; P=3.4e-07). See Supplementary Table 1 and Figure 2.

**Figure 3:**
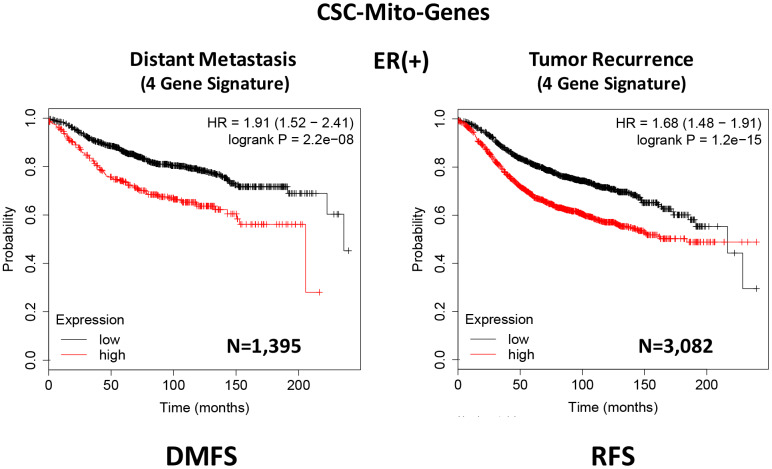
**A CSC-based mitochondrial 4-gene signature predicts distant metastasis and tumor recurrence in ER(+) breast cancer patients.** To optimize its predictive value, we constructed a short 4-gene signature, which revealed an increase in prognostic value, related to distant metastasis (HR=1.91; P=2.2e-08). This 4-gene signature was also able to predict tumor recurrence in the same patient population (HR=1.68; P=1.2e-15). See also Supplementary Tables 2 and 3 and 3. Therefore, these CSC-based mitochondrial signatures may provide a new prognostic approach for predicting treatment failure in breast cancer patients. DMFS, distant metastasis free survival; RFS, relapse free survival.

To optimize its predictive value, we next selected the top 4 gene transcripts, with the largest hazard ratios, to construct a short 4-gene signature, which revealed an increase in prognostic value, related to distant metastasis (HR=1.91; P=2.2e-08). Remarkably, this 4-gene signature was also able to predict tumor recurrence in the same patient population (HR=1.68; P=1.2e-15; Supplementary Table 2 and Figure 3).

Therefore, we conclude that these CSC-based mitochondrial signatures may provide a new prognostic approach for predicting distant metastasis and tumor recurrence in breast cancer patients. Most importantly, these results may also biologically and functionally implicate CSC mitochondria in the process of metastasis and tumor recurrence.

### Mitochondrial inhibitors metabolically target and prevent cancer cell metastasis, without significant toxicity

**Figure 4:**
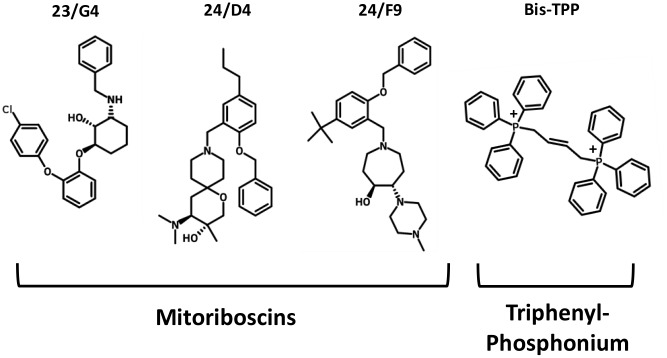
**Mitochondrial inhibitors: Mitoriboscins and bis-1,4-butene-TPP.** The chemical structures of the three Mitoriboscins (23/G4, 24/D4 and 24/F9) and Bis-TPP are shown.

To functionally evaluate the role of mitochondria in cancer metastasis, we used a series of mitochondrial inhibitors that were previously developed to specifically target the propagation of CSCs, known as the Mitoriboscins [15]. These inhibitors were developed via *in silico* screening of a library of 45,000 compounds, to identify positive hits that bound to the 3D-structure of the large mitochondrial ribosome [15]. After 880 positive hits were identified, these compounds were then subjected to phenotypic drug screening, using an ATP-depletion assay, and directly validated using the Seahorse Metabolic Flux analyser, to confirm their specificity as *bonafide* mitochondrial inhibitors [15]. Ultimately, this screening approach led to the identification of three major compounds, known as 23/G4, 24/D4 and 24/F9, which all inhibited 3D-mammosphere formation in MCF7 cells and significantly blocked cell migration in MDA-MB-231 cells, all in the low micro-molar range [15]. The structures of 23/G4, 24/D4 and 24/F9 are shown in Figure 4.

To experimentally evaluate their functional effects *in vivo*, we used MDA-MB-231 cells and the well-established chorio-allantoic membrane (CAM) assay in chicken eggs, to quantitatively measure tumor growth and distant metastasis. An inoculum of 1 X 10^6^ MDA-MB-231 cells was added onto the CAM of each egg (day E9) and then eggs were randomized into groups. On day E10, tumors were detectable and they were then treated daily for 8 days with vehicle alone (1% DMSO in PBS) or the three Mitoriboscin compounds. In parallel, we also evaluated the activity of another mitochondrial inhibitor, namely butane-1,4-bis-triphenyl-phosphonium (Bis-TPP), which we identified as an inhibitor of 3D-mammosphere formation in MCF7 cells, with an IC-50 of less than 0.5 μM [16]. It is well-established that the TPP-moiety acts as a chemical signal for mitochondrial targeting [16, 17].

After 8 days of drug administration, on day E18 all tumors were weighed, and the lower CAM was collected to evaluate the number of metastatic cells, as analyzed by qPCR with specific primers for Human Alu sequences.

**Figure 5:**
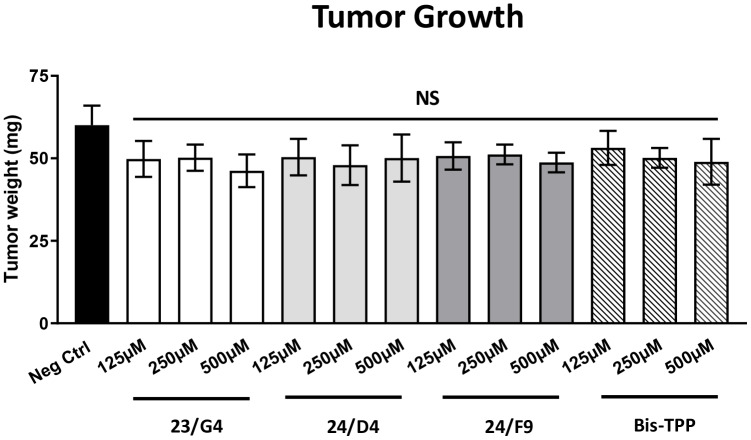
**Mitochondrial inhibitors have no effect on tumor growth.** MDA-MB-231 cells and the well-established chorio-allantoic membrane (CAM) assay in chicken eggs were used to quantitatively measure tumor growth. An inoculum of 1 X 10^6^ MDA-MB-231 cells was added onto the CAM of each egg (on Day E9) and then eggs were then randomized into groups. On day E10, tumors were detectable and they were then treated daily for 8 days with vehicle alone (1% DMSO in PBS) or the four mitochondrial inhibitors. After 8 days of drug administration, on day E18 all tumors were weighed. Note that none of the mitochondrial inhibitors tested had any significant effects on tumor growth. Averages are shown + SEM. NS, not significant.

Figure 5 shows the effects of the three Mitoriboscins (23/G4, 24/D4, 24/F9) and Bis-TPP on MDA-MB-231 tumor growth. Note that none of the four inhibitors tested showed any significant effects on tumor growth, as a result of the 8-day period of drug administration.

**Figure 6:**
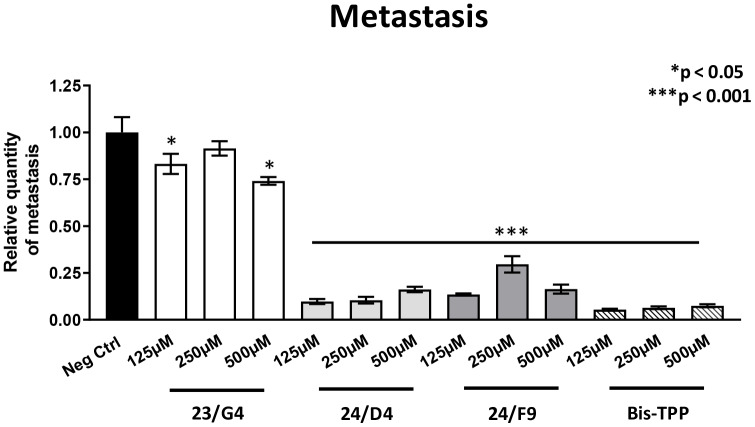
**Mitochondrial inhibitor compounds selectively target and prevent cancer metastasis.** MDA-MB-231 cells and the well-established chorio-allantoic membrane (CAM) assay in chicken eggs were used to quantitatively measure spontaneous tumor mestastasis. An inoculum of 1 X 10^6^ MDA-MB-231 cells was added onto the CAM of each egg (on day E9) and then eggs were then randomized into groups. On day E10, tumors were detectable and they were then treated daily for 8 days with vehicle alone (1% DMSO in PBS) or the four mitochondrial inhibitors. After 8 days of drug administration, the lower CAM was collected to evaluate the number of metastatic cells, as analyzed by qPCR with specific primers for Human Alu sequences. Note that all four mitochondrial inhibitors did show significant effects on MDA-MB-231 metastasis. More specifically, all three Mitoriboscins were clearly effective in inhibiting metastasis, although 24/D4 and 24/F9 were the most effective. In addition, Bis-TPP also significantly prevented metastasis. Averages are shown + SEM. *p<0.05; ***p<0.001.

However, all four mitochondrial inhibitors showed significant effects on MDA-MB-231 cancer cell metastasis. Figure 6 illustrates that all three Mitoriboscins were clearly effective in inhibiting metastatic progression, although 24/D4 and 24/F9 were the most effective. In addition, Bis-TPP also significantly prevented metastasis.

**Figure 7:**
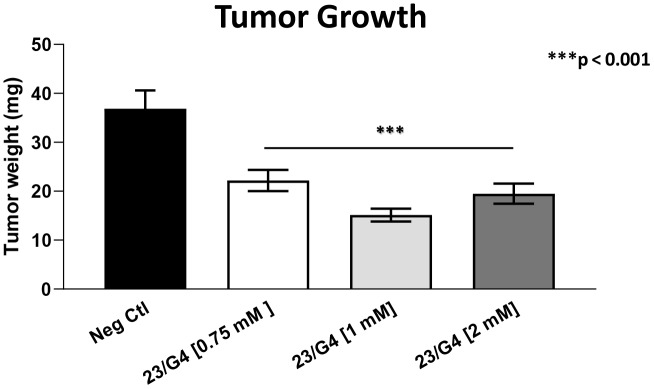
**Effects of the Mitoriboscin 23/G4 on tumor growth.** The Mitoriboscin 23/G4 was tested at higher concentrations of 0.75, 1 and 2 mM. Note that 23/G4, at these concentrations, inhibited tumor growth (by 40% to 60%). Averages are shown + SEM. ***p<0.001.

**Figure 8:**
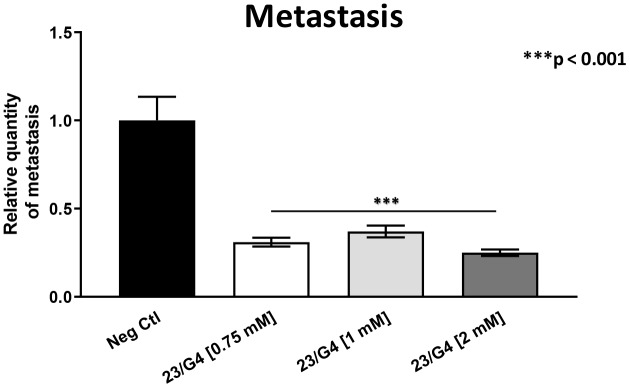
**Effects of the Mitoriboscin 23/G4 on cancer metastasis.** The Mitoriboscin 23/G4 was tested at higher concentrations, namely 0.75, 1 and 2 mM. Note that 23/G4, at these concentrations, significantly inhibited metastasis (by 70-75%). Interestingly, the effects of 23/G4 on metastasis were significantly more pronounced than its effects on tumor growth. Averages are shown + SEM. ***p<0.001.

As 23/G4 was minimally effective at a concentration of 0.5 mM, we also tested it at higher concentrations of 0.75, 1 and 2 mM. Importantly, our results show that 23/G4, at these concentrations, significantly inhibited both tumor growth (by 40% to 60%; Figure 7) and metastatic progression (by 70-75%; Figure 8). Interestingly, as expected, the effects of 23/G4 on metastasis were significantly more pronounced.

Remarkably, in this series of experiments, little or no embryo toxicity was observed, otherwise tumor growth and cancer metastasis assays could not have been completed (Tables 1–3). Therefore, we conclude that mitochondrial inhibitors can be used experimentally, to preferentially inhibit the initiation of tumor metastasis, without significant toxicity.

**Table 1:** Chick embryo toxicity of Mitoriboscins and Bis-TPP at a concentration of 0.5 mM.

**Group #**	**Group Description**	**Total**	**Alive**	**Dead**	**% Alive**	**% Dead**
**1**	Neg. Ctrl.	18	16	2	88.89	11.11
**2**	23/G4	10	7	3	70	30
**3**	24/D4	12	12	0	100	0
**4**	24/F9	10	8	2	80	20
**5**	Bis-TPP	12	7	5	58.33	41.67

**Table 2:** Chick embryo toxicity of Mitoriboscin 23/G4 at higher concentrations.

**Group #**	**Group Description**	**Total**	**Alive**	**Dead**	**% Alive**	**% Dead**
**1**	Neg Ctrl	17	12	5	70.59	29.41
**2**	23/G4 [0.75 mM]	14	10	4	71.43	28.57
**3**	23/G4 [1 mM]	15	12	3	80	20
**4**	23/G4 [2 mM]	15	10	5	66.67	33.33

**Table 3:** Chick embryo toxicity of Dodecyl-TPP.

**Group #**	**Group Description**	**Total**	**Alive**	**Dead**	**% Alive**	**% Dead**
**1**	Neg. Ctrl.	18	13	5	72.22	27.78
**2**	d-TPP [6.25 μM]	19	13	6	68.42	31.58
**3**	d-TPP [25 μM]	19	13	6	68.42	31.58
**4**	d-TPP [62.5 μM]	19	3	16	15.79	84.21

**Figure 9:**
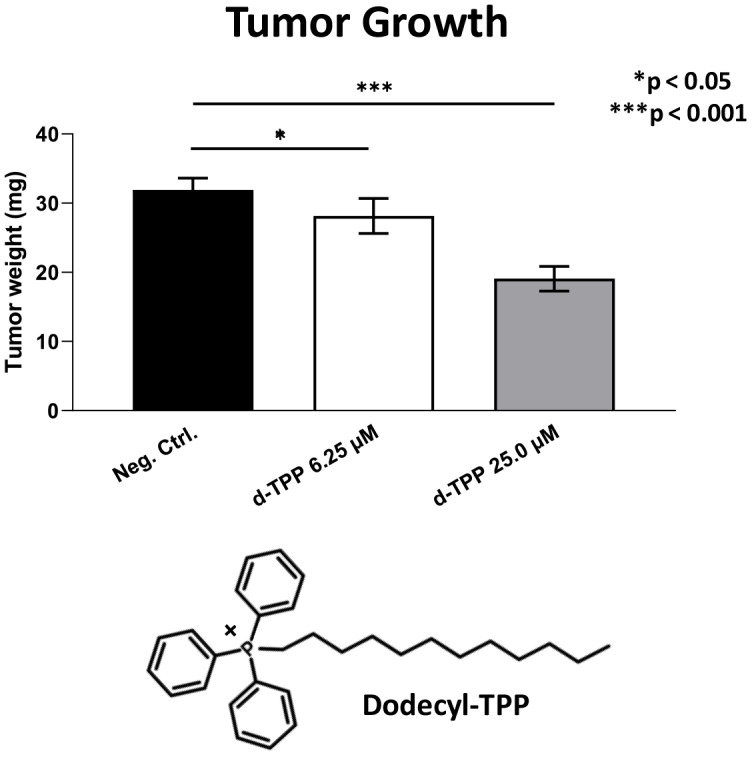
**Effects of Dodecyl-TPP on tumor growth.** Dodecyl-TPP, another more potent mitochondrially-targeted TPP compound, was tested using low micro-molar concentrations (6.25- and 25-μM). Note that Dodecyl-TPP significantly inhibited tumor growth (by 12% to 40%). Averages are shown + SEM. *p<0.05; ***p<0.001. The structure of Dodecyl-TPP (d-TPP) is also shown. Note the 12-carbon alkyl-chain attached to the lipophilic cation, triphenyl-phosphonium (TPP).

**Figure 10:**
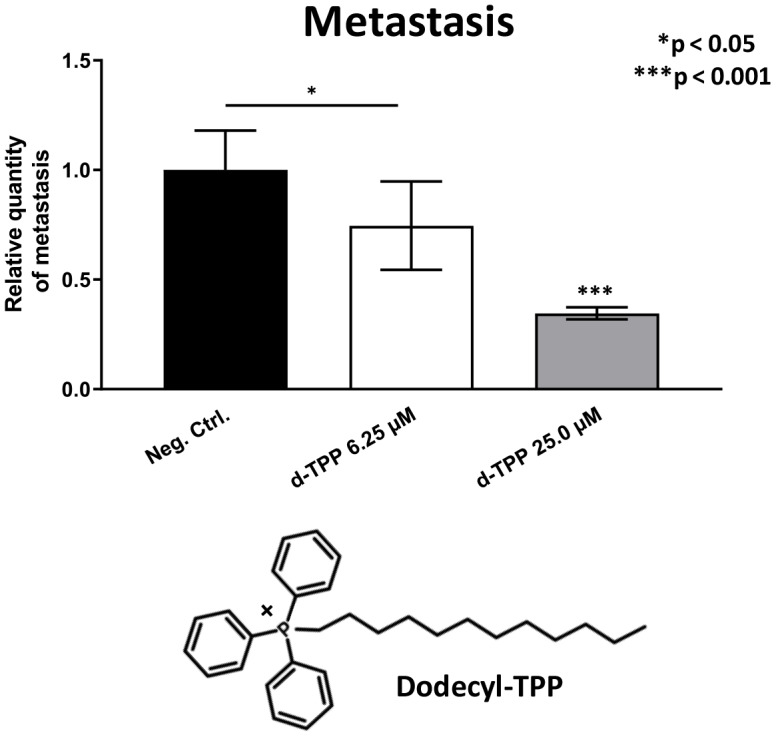
**Effects of Dodecyl-TPP on cancer metastasis.** Dodecyl-TPP was tested using low micro-molar concentrations (6.25- and 25-μM). Note that Dodecyl-TPP significantly inhibited metastasis (by 25% to 65%). Averages are shown + SEM. *p<0.05; ***p<0.001. Importantly, little or no toxicity was observed for Dodecyl-TPP at 6.25- and 25-μM (Table 3). The structure of Dodecyl-TPP (d-TPP) is also shown.

Finally, we also tested another more potent mitochondrially-targeted TPP compound, namely Dodecyl-TPP, using low micro-molar concentrations (6.25- and 25-μM). Figures 9 and 10 demonstrate that Dodecyl-TPP significantly inhibited tumor growth (by 12% to 40%; Figure 9) and metastatic progression (by 25 to 65%; Figure 10). As predicted, Dodecyl-TPP preferentially targeted metastasis, rather than tumor growth. It is worth noting that Dodecyl-TPP showed some toxicity, but only at 62.5-μM, preventing reliable analysis of its effects on tumor growth and metastasis, at this higher concentration (Table 3). However, Dodecyl-TPP showed little or no toxicity at 6.25- and 25-μM (Table 3).

### Mito-Ribosome based signatures for predicting distant metastasis and tumor recurrence: implications as companion diagnostics

Given the functional effects of the Mitoriboscin compounds on metastasis, we next evaluated if the gene mRNA transcripts of the large mitochondrial ribosomal proteins (MRPL) show any prognostic value in ER(+) and ER(-)/basal breast cancer patients.

**Figure 11:**
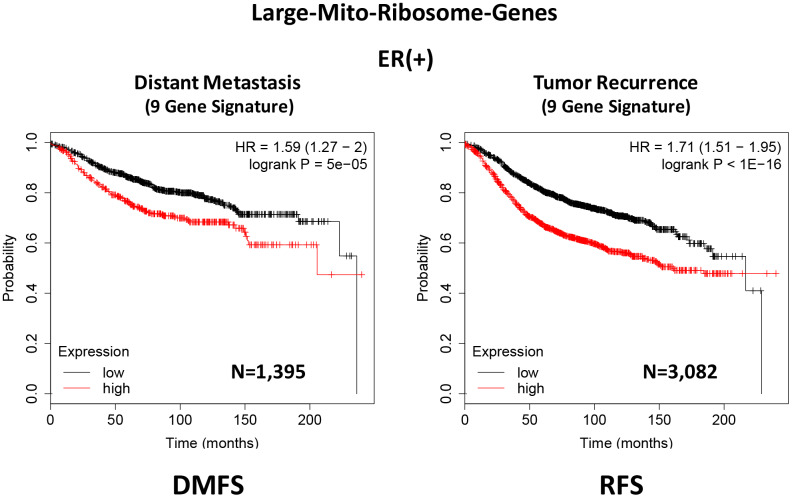
**A large mito-ribosome 9-gene signature predicts distant metastasis and tumor recurrence in ER(+) breast cancer patients.** A 9-gene mito-ribosome signature effectively predicts distant metastasis in N=1,395 patients (HR=1.59; P=5e-05) and tumor recurrence in N=3,082 patients (HR=1.71; P<1e-16). See Supplementary Tables 4 and 5 and 5 DMFS, distant metastasis free survival; RFS, relapse free survival.

**Figure 12:**
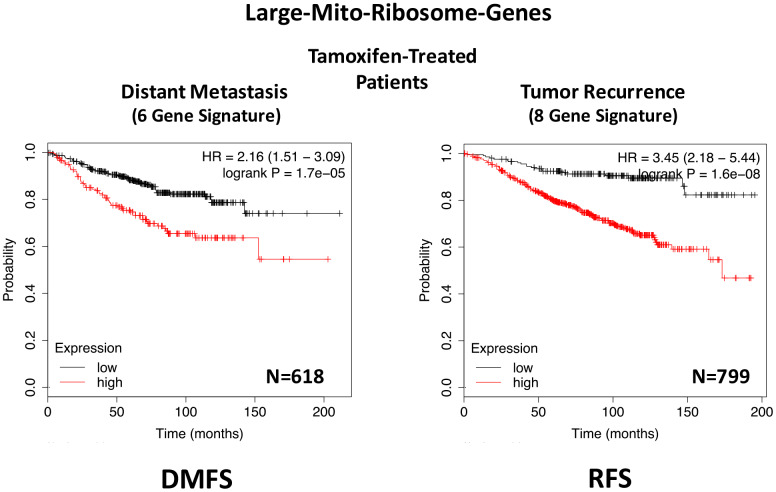
**A large mito-ribosome gene signature predicts distant metastasis and tumor recurrence in ER(+) breast cancer patients, treated with Tamoxifen.** A mito-ribosome signature predicts treatment failure in a sub-set of ER(+) patients undergoing Tamoxifen treatment, which resulted in distant metastasis (N=618 patients; HR=2.16; P=1.7e-05) and tumor recurrence (N=799 patients; HR=3.45; P=1.6e-08). See also Supplementary Tables 6 and 7 and [Supplementary-material SD2]. DMFS, distant metastasis free survival; RFS, relapse free survival.

In ER(+) breast cancer, a 9-gene mito-ribosome signature was able to effectively predict distant metastasis in N=1,395 patients (HR=1.59; P=5e-05) and tumor recurrence in N=3,082 patients (HR=1.71; P<1e-16) (See Supplementary Tables 4 and 5; Figure 11). Importantly, a closely related mito-ribosome signature was also able to predict treatment failure in a sub-set of ER(+) patients undergoing Tamoxifen treatment, which resulted in distant metastasis (N=618 patients; HR=2.16; P=1.7e-05) and tumor recurrence (N=799 patients; HR=3.45; P=1.6e-08) (Supplementary Tables 6 and 7; Figure 12).

**Figure 13:**
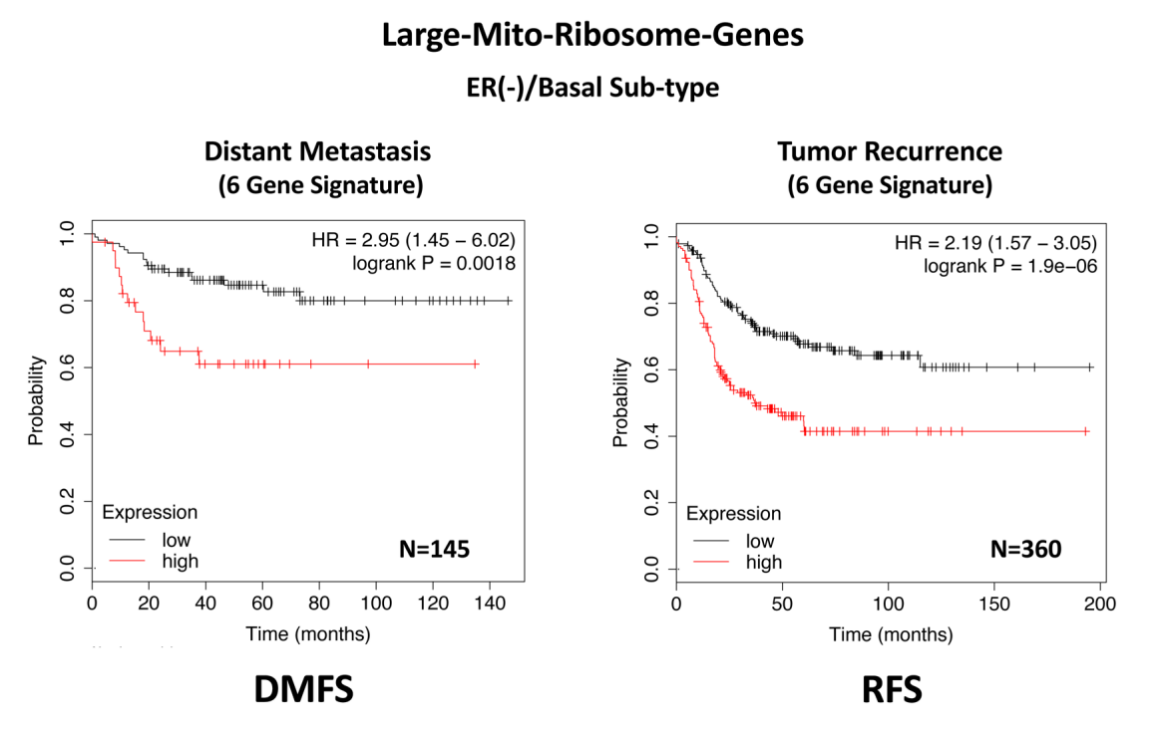
**A large mito-ribosome gene signature predicts distant metastasis and tumor recurrence in ER(-)/basal breast cancer patients.** In ER(-)/basal breast cancer, a 6-gene mito-ribosome signature was also able to effectively predict distant metastasis in N=145 patients (HR=2.95; P=0.0018) and tumor recurrence in N=360 patients (HR=2.19; P=1.9e-06). See also Supplementary Table 8.

**Figure 14:**
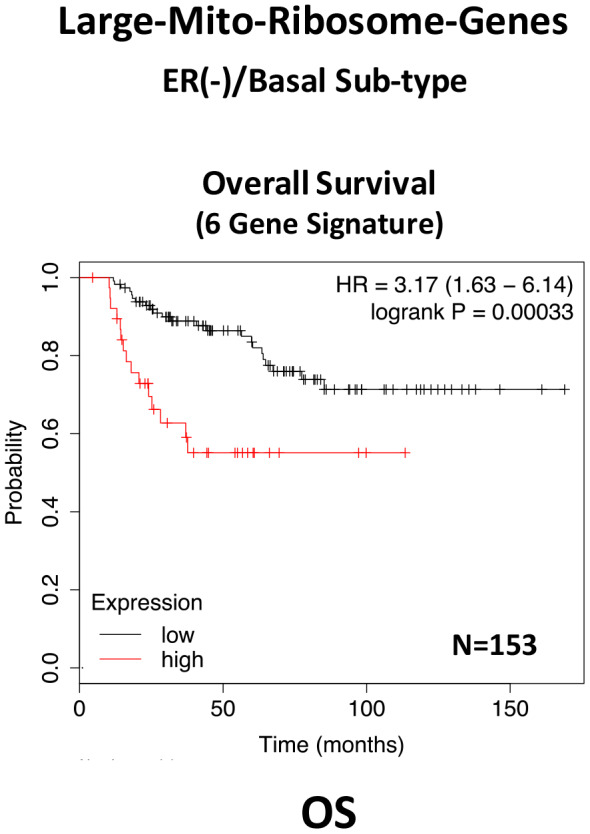
**A large mito-ribosome gene signature predicts overall survival in ER(-)/basal breast cancer patients.** In ER(-)/basal breast cancer, a 6-gene mito-ribosome signature was also able to effectively predict overall survival in N=153 patients (HR=3.17; P=0.00033).

In ER(-)/basal breast cancer, a distinct 6-gene mito-ribosome MRPL signature was also able to effectively predict distant metastasis in N=145 patients (HR=2.95; P=0.0018) and tumor recurrence in N=360 patients (HR=2.19; P=1.9e-06), as well as overall survival in N=153 patients (HR=3.17; P=0.00033) (Supplementary Table 8; Figures 13 and 14).

In summary, these short mito-ribosome gene signatures may also be useful as companion diagnostics to assess which patient populations may benefit most from the administration of the Mitoriboscin compounds.

## DISCUSSION

Current thinking indicates that CSCs are the etiological cause of treatment failure in most cancer patients, as they are the cellular drivers of tumor recurrence, metastasis and drug-resistance [1–5]. As a consequence, new therapeutic approaches are needed to effectively eliminate CSCs. Our previous studies identified CSC mitochondria as a potential new therapeutic target. More specifically, we experimentally observed that MCF7-derived 3D-mammospheres are specifically enriched in mitochondrial proteins; 25 mitochondrial proteins showed greater than 100-fold over-expression, while 9 of these proteins were infinitely up-regulated, as compared with 2D-monolayers [6]. In this report, we used these proteomic data as possible candidates to generate short mitochondrial gene signatures that could be employed as prognostic tools to predict distant metastasis (in N=1,395 patients) and tumor recurrence (in N=3,082 patients), in a large collection of ER(+) breast cancer patients. For example, we developed a 4-gene signature for predicting distant metastasis, resulting in a hazard ratio of 1.91-fold (P=2.2e-08). This clinical evidence supports the idea that CSC mitochondria may play a critical functional role in the metastatic dissemination of cancer cells.

**Figure 15:**
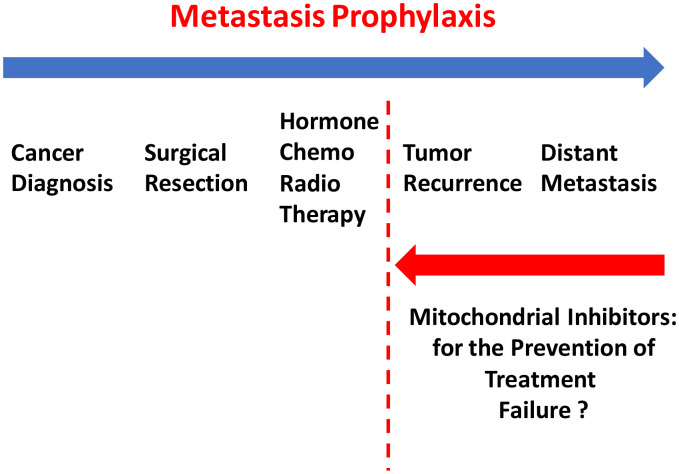
**Metastasis prophylaxis: clinical implications of mitochondrial inhibitors for the prevention of treatment failure and cancer metastasis.** Based on our current results, that mitochondrial inhibitors can selectively prevent metastasis, we suggest that these findings could be applied clinically to help prevent treatment failure in breast cancer patients.

To further test this hypothesis experimentally, we next employed a well-established animal model, namely the chorio-allantoic membrane (CAM) in chicken eggs, to test a series of mitochondrial inhibitors. These mitochondrial inhibitors, including the Mitoriboscins, have been previously described to effectively inhibit 3D-mammosphere formation in MCF7 cells and cell migration in MDA-MB-231 cells. All five of these mitochondrial inhibitors selectively prevented MDA-MB-231 tumor cell metastasis, but had only minor effects or no effect on tumor formation. More specifically, we have previously shown that these mitochondrial inhibitors successfully induce ATP-depletion in cancer cells, by targeting mitochondrial protein translation and/or OXPHOS activity [15–17]. Our current studies provide the necessary *in vivo* functional evidence, that mitochondrial inhibitors can successfully prevent cancer metastasis. These findings could have important clinical implications, for ultimately preventing treatment failure in breast cancer patients, via metastasis prophylaxis (Figure 15).

Since the Mitoriboscins were originally engineered to inhibit the large mitochondrial ribosome [15], we also focused on whether the large mitochondrial ribosomal gene transcripts (MRPL) have any prognostic value, for predicting distant metastasis in ER(+) breast cancer patients. Importantly, signatures containing MRPL gene transcripts were effective in predicting metastasis, recurrence and Tamoxifen-resistance. Similar results were also obtained in ER(-) breast cancer patients. As a consequence of the success of this approach, these MRPL gene signatures may ultimately be useful as new companion diagnostics, to guide decisions to determine which patients would benefit from anti-mito-ribosome therapy.

## MATERIALS AND METHODS

### Materials

MDA-MB-231 cells, a human breast cancer cell line, were obtained from the American Type Culture Collection (ATCC). Mitoriboscins (23/G4, 24/D4, 24/F9), Butene-1,4-bis-triphenyl-phosphonium (Bis-TPP), and Dodecyl-TPP, were all as we previously described [15–17].

### Kaplan-Meier (K-M) analyses

To perform K-M analysis on gene transcripts, we used an open-access online survival analysis tool to interrogate publically available microarray data from up to 3,951 breast cancer patients (18). This allowed us to determine their prognostic value. For this purpose, we primarily analyzed data from ER(+)s and ER(-)/basal patients. Biased array data were excluded from the analysis. This allowed us to identify mitochondrial gene transcripts, with significant prognostic value. Hazard-ratios were calculated, at the best auto-selected cut-off, and p-values were calculated using the Log-rank test and plotted in R. K-M curves were also generated online using the K-M-plotter (as high-resolution TIFF files), using univariate analysis: https://kmplot.com/analysis/index.php?p=service&cancer=breast.

This approach allowed us to directly perform *in silico* validation of these mitochondrial biomarker candidates. The multi-gene classifier function of the program was used to test the prognostic value of short mitochondrial gene signatures, using the mean expression of the selected probes. The latest 2020 version of the database was utilized for all these analyses.

### Assays for tumor growth, metastasis and embryo toxicity

### Preparation of chicken embryo

Fertilized White Leghorn eggs were incubated at 37.5°C with 50% relative humidity for 9 days. At that moment (E9), the chorioallantoic membrane (CAM) was dropped down by drilling a small hole through the eggshell into the air sac, and a 1 cm² window was cut in the eggshell above the CAM (19-23).

### Amplification and grafting of tumor cells

The MDA-MB-231 tumor cell line was cultivated in DMEM medium supplemented with 10% FBS and 1% penicillin/streptomycin. On day E9, cells were detached with trypsin, washed with complete medium and suspended in graft medium. An inoculum of 1 X 10^6^ cells was added onto the upper CAM of each egg (E9) and then eggs were randomized into groups [19–23].

### Tumor growth assays

At day 18 (E18), the upper portion of the CAM was removed from each egg, washed in PBS and then directly transferred to paraformaldehyde (fixation for 48 h) and weighed [19–23]. For tumor growth assays, at least 8 tumor samples were collected and analysed per group (n > 8).

### Metastasis assays

On day E18, a 1 cm² portion of the lower CAM was collected to evaluate the number of metastatic cells in 8 samples per group (n=8). Genomic DNA was extracted from the CAM (commercial kit) and analyzed by qPCR with specific primers for Human Alu sequences. Calculation of Cq for each sample, mean Cq and relative amounts of metastases for each group are directly managed by the Bio-Rad® CFX Maestro software. A one-way ANOVA analysis with post-tests was performed on all the data [19–23].

### Embryo tolerability assay

Before each administration, the treatment tolerability was evaluated by scoring the number of dead embryos. This approached is summarized schematically in Supplementary Figures 1 and 2.

## Supplementary Material

Supplementary Figures

Supplementary Tables
