# Supplementary material for: First-in-class candidate therapeutics that target mitochondria and effectively prevent cancer cell metastasis: mitoriboscins and TPP compounds
Source: Aging (Albany NY). 2020 May 24;12(11):10162–79. doi: 10.18632/aging.103336 (PMC7346015; doi:10.18632/aging.103336)
Supplement: Supplementary Figures [file aging-12-103336-s002..pdf]

SUPPLEMENTARY FIGURES

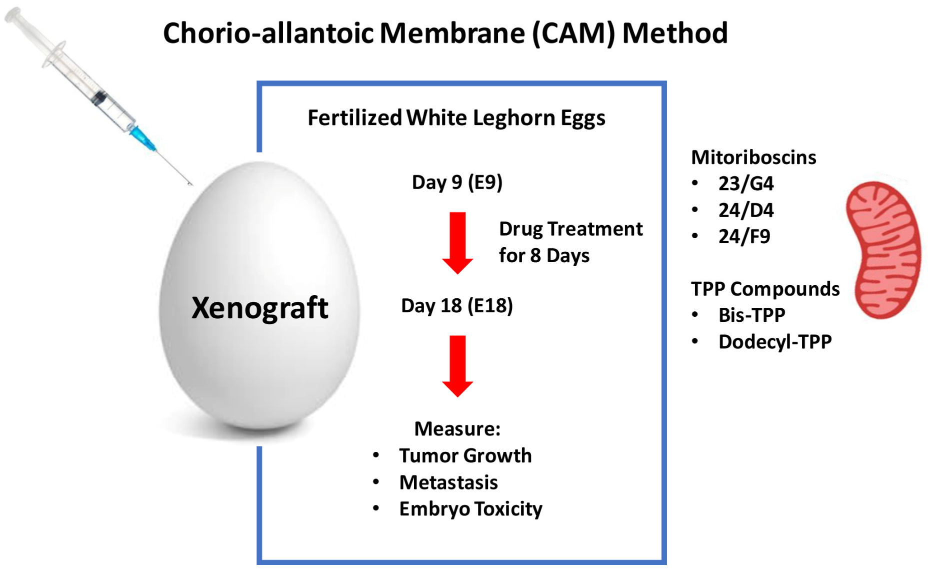

Supplementary Figure 1. Summary: The Chorio-allantoic Membrane (CAM) Method. Fertilized White Leghorn eggs were as used the xenograft host for growing human MDA-MB-231 cells to measure i) tumor growth (weight), ii) metastatic invastion/progression (by qPCR with specific primers for Human Alu sequences) and iii) drug toxicity (by evaluating chicken embryo viability). MDA-MB-231 cells were implanted on embryonic day 9 (E9). Then, tumor growth and metastasis were evaluated on embryonic day 18 (E18). Treatments were administered for 8 days, from E10 till E17. Using this approach, the efficacy and toxicity of five mitochondrial inhibitors were evaluated, in an *in vivo* setting: Mitoriboscins (23/G4, 24/D4, 24/F9) and TPP compounds (Bis-TPP and Dodecyl-TPP). See *Materials & Methods* for specific details.

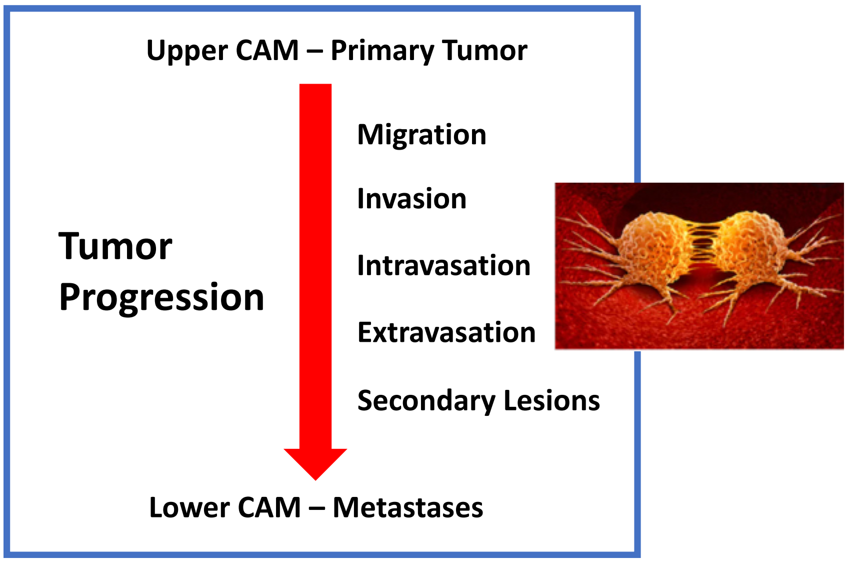

Supplementary Figure 2. Measuring Distant Metastasis with the CAM Method. Spontaneous metastasis from the primary tumor located in the Upper CAM was measured by quantitating the amount of metastatic cells that accumulated in the Lower CAM region, after a period of 9 days, post-tumor cell implantation, using qPCR analysis. Importantly, metastatic dissemination, from the Upper CAM to the Lower CAM, requires cell migration, invasion, intravasation, extravasation and the formation of secondary lesions.
