# Supplementary material for: First-in-class candidate therapeutics that target mitochondria and effectively prevent cancer cell metastasis: mitoriboscins and TPP compounds
Source: Aging (Albany NY). 2020 May 24;12(11):10162–79. doi: 10.18632/aging.103336 (PMC7346015; doi:10.18632/aging.103336)
Supplement: Supplementary Tables [file aging-12-103336-s001..pdf]

SUPPLEMENTARY TABLES

Supplementary Table 1. Distant metastasis signature (13 genes): prognostic palue of mitochondrial-related proteins up-regulated in MCF7 mammospheres, evaluated in ER(+) breast cancer patients (DMFS/ER(+)/N=1,395/>240-months).

Probe	Symbol	HR (DMFS)	Log-rank test
201128_s_at	ACLY	1.72	3.1e-05
208845_at	VDAC3	1.66	1.2e-05
202282_at	HADH2	1.58	7.2e-05
201441_at	COX6B1	1.53	0.00017
201322_at	ATP5B	1.43	0.0016
218440_at	MCCC1	1.40	0.0054
218275_at	SLC25A10	1.37	0.0048
205217_at	TIMM8A	1.37	0.0072
200789_at	ECH1	1.35	0.0092
212186_at	ACACA	1.34	0.031
200690_at	HSPA9	1.29	0.046
217720_at	CHCHD2	1.28	0.028
217814_at	CCDC47	1.28	0.032
**Combined**		**1.79**	**3.4e-07**

Supplementary Table 2. Distant metastasis signature (4 genes): prognostic value of mitochondrial-related proteins up-regulated in MCF7 mammospheres, evaluated in ER(+) breast cancer patients (DMFS/ER(+)/N=1,395/>240-months).

Probe	Symbol	HR (DMFS)	Log-rank test
201128_s_at	ACLY	1.72	3.1e-05
208845_at	VDAC3	1.66	1.2e-05
202282_at	HADH2	1.58	7.2e-05
201441_at	COX6B1	1.53	0.00017
**Combined**		**1.91**	**2.2e-08**

Supplementary Table 3. Tumor recurrence signature (4 genes): prognostic value of mitochondrial-related proteins up-regulated in MCF7 mammospheres, evaluated in ER(+) breast cancer patients (RFS/ER(+)/N=3,082/>240-months).

Probe	Symbol	HR (RFS)	Log-rank test
208845_at	VDAC3	1.56	2.3e-11
202282_at	HADH2	1.52	1.3e-09
201441_at	COX6B1	1.51	1.3e-10
201128_s_at	ACLY	1.12	0.091
**Combined**		**1.68**	**1.2e-15**

Supplementary Table 4. Distant metastasis signature (9 genes): prognostic value of large mito-ribosomal proteins, evaluated in ER(+) breast cancer patients (DMFS /ER(+)/N=1,395/>240-months).

Probe	Symbol	HR (DMFS)	Log-rank test
218027_at	MRPL15	1.68	4.1e-06
218049_s_at	MRPL13	1.56	8.8e-05
222216_s_at	MRPL17	1.53	0.00044
219244_s_at	MRPL46	1.46	0.0013
217907_at	MRPL18	1.40	0.005
218281_at	MRPL48	1.38	0.0078
208787_at	MRPL3	1.37	0.0086
218270_at	MRPL24	1.33	0.021
218105_s_at	MRPL4	1.29	0.023
**Combined**		**1.59**	**5e-05**

Supplementary Table 5. Tumor recurrence signature (9 genes): prognostic value of large mito-ribosomal proteins, evaluated in ER(+) breast cancer patients (RFS /ER(+)/N=3,082/>240-months).

Probe	Symbol	HR (RFS)	Log-rank test
218027_at	MRPL15	1.72	<1e-16
218049_s_at	MRPL13	1.71	<1e-16
208787_at	MRPL3	1.71	<1e-16
222216_s_at	MRPL17	1.70	1.1e-16
217907_at	MRPL18	1.55	2e-11
218281_at	MRPL48	1.26	0.00041
218105_s_at	MRPL4	1.24	0.00095
219244_s_at	MRPL46	1.21	0.0083
218270_at	MRPL24	1.10	0.2
**Combined**		**1.71**	**<1e-16**

Supplementary Table 6. Distant metastasis signature (6 genes): prognostic value of large mito-ribosomal proteins, evaluated in ER(+) breast cancer patients (DMFS /ER(+)/N=618/>240-months) treated with Tamoxifen.

Probe	Symbol	HR (DMFS)	Log-rank test
218027_at	MRPL15	2.15	1.7e-06
219244_s_at	MRPL46	1.99	0.00011
222216_s_at	MRPL17	1.99	0.0036
218270_at	MRPL24	1.94	0.00024
217907_at	MRPL18	1.71	0.0051
218049_s_at	MRPL13	1.55	0.021
**Combined**		**2.16**	**1.7e-05**

Supplementary Table 7. Tumor recurrence signature (8 genes): prognostic value of large mito-ribosomal proteins, evaluated in ER(+) breast cancer patients (RFS /ER(+)/N=799/>240-months) treated with Tamoxifen.

Probe	Symbol	HR (RFS)	Log-rank test
218027_at	MRPL15	2.20	7.8e-08
208787_at	MRPL3	2.04	0.00015
222216_s_at	MRPL17	2.01	2.3e-06
217907_at	MRPL18	1.92	9.1e-06
218270_at	MRPL24	1.77	0.00012
218049_s_at	MRPL13	1.66	0.0059
218281_at	MRPL48	1.55	0.0033
219244_s_at	MRPL46	1.53	0.0063
**Combined**		**3.45**	**1.6e-08**

Supplementary Table 8. Distant metastasis signature (6 genes): prognostic value of large mito-ribosomal proteins, evaluated in ER(-)/basal breast cancer patients (DMFS/N=145/>120-months).

Probe	Symbol	HR (DMFS)	Log-rank test
222466_s_at	MRPL42	4.00	0.0024
227186_s_at	MRPL41	3.43	0.0023
225797_at	MRPL54	2.44	0.011
218049_s_at	MRPL13	2.23	0.0022
224331_s_at	MRPL36	2.13	0.036
218339_at	MRPL22	1.75	0.029
**Combined**		**2.95**	**0.0018**
